# Okadaic Acid Exposure Induced Neural Tube Defects in Chicken (*Gallus gallus*) Embryos

**DOI:** 10.3390/md19060322

**Published:** 2021-06-02

**Authors:** Yuhu Jiao, Guang Wang, Dawei Li, Hongye Li, Jiesheng Liu, Xuesong Yang, Weidong Yang

**Affiliations:** 1 Key Laboratory of Aquatic Eutrophication and Control of Harmful Algal Blooms of Guangdong Higher Education Institute, College of Life Science and Technology, Jinan University, Guangzhou 510632, China; jiaoyuhu0809@stu.jnu.edu.cn (Y.J.); daweili@jnu.edu.cn (D.L.); thyli@jnu.edu.cn (H.L.); tjsliu@jnu.edu.cn (J.L.); 2 Key Laboratory for Regenerative Medicine of the Ministry of Education, Division of Histology and Embryology, Medical College, Jinan University, Guangzhou 510632, China; t_wangguang@jnu.edu.cn

**Keywords:** okadaic acid, chick embryo model, NTDs, Toll-like signaling pathway

## Abstract

Okadaic acid (OA) is an important liposoluble shellfish toxin distributed worldwide, and is mainly responsible for diarrheic shellfish poisoning in human beings. It has a variety of toxicities, including cytotoxicity, embryonic toxicity, neurotoxicity, and even genotoxicity. However, there is no direct evidence of its developmental toxicity in human offspring. In this study, using the chicken (*Gallus gallus*) embryo as the animal model, we investigated the effects of OA exposure on neurogenesis and the incidence of neural tube defects (NTDs). We found that OA exposure could cause NTDs and inhibit the neuronal differentiation. Immunofluorescent staining of pHI3 and c-Caspase3 demonstrated that OA exposure could promote cell proliferation and inhibit cell apoptosis on the developing neural tube. Besides, the down-regulation of Nrf2 and increase in reactive oxygen species (ROS) content and superoxide dismutase (SOD) activity in the OA-exposed chicken embryos indicated that OA could result in oxidative stress in early chick embryos, which might enhance the risk of the subsequent NTDs. The inhibition of bone morphogenetic protein 4 (BMP4) and Sonic hedgehog (Shh) expression in the dorsal neural tube suggested that OA could also affect the formation of dorsolateral hinge points, which might ultimately hinder the closure of the neural tube. Transcriptome and qPCR analysis showed the expression of lipopolysaccharide-binding protein (LBP), transcription factor AP-1 (JUN), proto-oncogene protein c-fos (FOS), and C-C motif chemokine 4 (CCL4) in the Toll-like receptor signaling pathway was significantly increased in the OA-exposed embryos, suggesting that the NTDs induced by OA might be associated with the Toll-like receptor signaling pathway. Taken together, our findings could advance the understanding of the embryo–fetal developmental toxicity of OA on human gestation.

## 1. Introduction

Okadaic acid (OA), an important marine toxin, is mainly responsible for diarrhetic shellfish poisoning (DSP) in human beings [1,2]. OA usually accumulates in the tissues of filter-feeding bivalves, and eventually pose a great threat to human health through the consumption of contaminated shellfish [3]. Obviously, OA has become a serious concern for the shellfish industry and public health since it is one of the most frequent and globally distributed marine biotoxins [2,4].

Previous studies have proved that OA is a potent and specific inhibitor of serine/threonine protein phosphatase 1 (PP1) and protein phosphatase 2A (PP2A) [5,6,7]. Studies show that OA has a variety of toxic effects, including cytotoxicity, carcinogenicity, neurotoxicity, as well as embryotoxicity [1,8]. OA is able to induce cell apoptosis in multiple human cell lines, such as TR14, NT2-N and SHSY5Y cells [9,10], malignant glioma cells [11], and HepaRG cells [12]. Interestingly, the nervous system is reported to be more sensitive to OA than other systems, though OA was not considered as a classical neurotoxin previously [9]. It has been demonstrated that OA can induce spatial memory impairment and neurodegeneration [13] and cause hippocampal cell loss in rats [14]. Due to its neurotoxicity, OA is judged to be an emerging tool for studies on Alzheimer’s disease [15,16].

In addition, OA has embryotoxic potential, and can delay the development of embryos and increase the incidence of malformation and mortality in the frog *Xenopus laevis* [17], the fish *Oryzias latipes* [18] and the chicken *Gallus gallus* [19]. Moreover, OA has been shown to cross the placental barrier in mice [20]. The content of OA in fetal tissues is even higher than that of adults, indicating the accumulation of OA in fetal tissue that causes even more damage to fetuses than adults [2,4]. Thus far, however, there has been no direct evidence about OA’s prenatal developmental toxicity on human beings [1].

As an important morphogenetic event in embryonic development, neurulation takes place in the early stage of chordate embryogenesis, and eventually, a closed neural tube is formed [21,22]. The failure of neural tube closure will cause a group of common and severe malformations known as neural tube defects (NTDs). As the second most prevalent malformations [22], NTDs affect more than 300,000 newborns worldwide each year [23]. The frequency of NTDs in pregnancies is about 1 per 1000. When NTDs occur in the head, anencephaly, hydrocephalus, encephalocele, it is frequently associated with other malformations. If NTDs are presented in the trunk, there is a greater chance of the occurrence of congenital defects such as spina bifida [24,25]. Therefore, based on the potential embryotoxicity of OA, it is of significance to evaluate the effect of OA exposure on embryonic neurogenesis.

The chicken (*G. gallus*) embryo is an excellent animal model and has been extensively used for studies of early vertebrate embryogenesis and late organogenesis [26]. As an in vivo experiment, the chicken embryo model has many advantages, such as convenience for observation, similarity to mammalian embryo, easy accessibility, and manipulation [27]. In this study, we employed the chick embryo as a model to explore the effects of OA exposure on embryonic neurogenesis and underlying mechanisms involved.

## 2. Results

### 2.1. OA Exposure Induced Craniofacial Abnormality in Early Stage Chick Embryos

**Figure 1:**
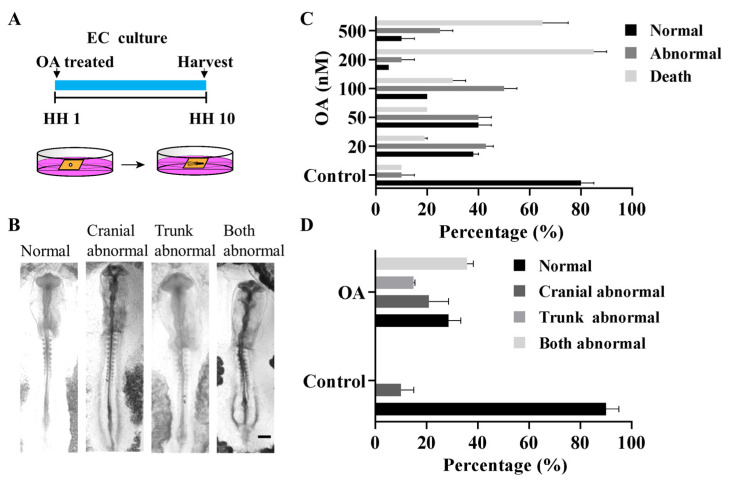
OA exposure induces neural tube defects in early chick embryos. (**A**) Diagram of early chick embryo culture in the presence of OA or PBS. (**B**) Phenotypes of neural tube defects after exposed to OA. (**C**) Incidences of mortality and abnormality of chick embryos exposed to different concentrations of OA (n = 3, 20 embryos per replicate). (**D**) NTD types and the percentages in OA-treated (100 nM) group (n = 3, 20 embryos per replicate). Scale bars = 200 μm in (**B**).

To explore the possible toxicological effects of OA on chick embryo development, EC culture was performed, as shown in Figure 1A. The HH10 chick embryos were exposed to the culture media containing different concentrations of OA (20, 50, 100, 200 and 500 nM). Three types of NTDs were observed, including cranial abnormality, trunk abnormality and both (Figure 1B), and some embryos died during the incubation. The mortality rate was the highest when embryos were exposed to OA at 200 nM or 500 nM, about 85% (Figure 1C), while the mortality rate was approximately 30% in the 20 nM or 50 nM OA treatment group (Figure 1C). When the embryo was exposed to OA at 100 nM, both cranial and trunk abnormality was about 40% (the highest) (Figure 1D). The embryos where the neural tubes were not closed on the cranial and trunk regions were selected as the research object.

### 2.2. OA Exposure Caused Craniofacial Abnormality in Late-stage Chick Embryos

**Figure 2:**
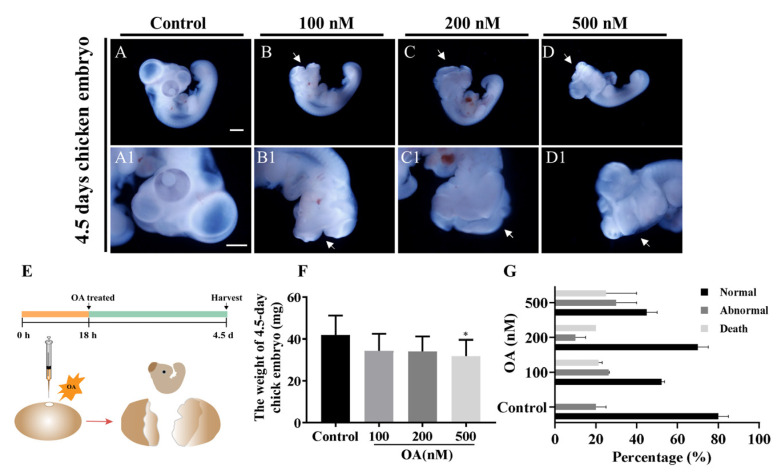
OA exposure induces neural tube defects in the late stage of chick embryos. (**A**–**D1**) OA exposure led to NTDs in 4.5-day chick embryo. (**E**) Diagram of late-stage chick embryo culture in the presence of OA or PBS. (**F**) The weight of 4.5-day chick embryo after exposure to OA (n = 3, 30 embryos replicates). (**G**) Incidences of mortality and abnormality of 4.5-day chick embryo after exposure to different concentrations of OA (n = 3, 20 embryos per replicate). Scale bars = 1 mm in (**A**–**D**), 1000 μm in A1–D1. One way ANOVA, * *p* < 0.05 indicate significant differences between the experimental and control groups.

After pre-incubation for 18 h, 100 μL of OA at concentrations of 100 nM, 200 nM and 500 nM were injected into the embryo, respectively (Figure 2E). After exposure to OA, we found some neural tube defects (NTDs) in 4.5-day chicken embryos (Figure 2A–D1). Compared with the control counterparts, the embryo mortality and malformation rate of embryos were increased (Figure 2G). In addition, the weight of the embryos showed a trend of decline after being exposed to OA. The weight was distinctly lower than that of the control when exposed to OA at 500 nM (*p* < 0.05) (Figure 2F,G). These results suggest that OA could cause neural tube defects in late-stage chick embryos.

### 2.3. OA Exposure Led to Abnormal Neurogenesis during Chick Embryo Development

**Figure 3:**
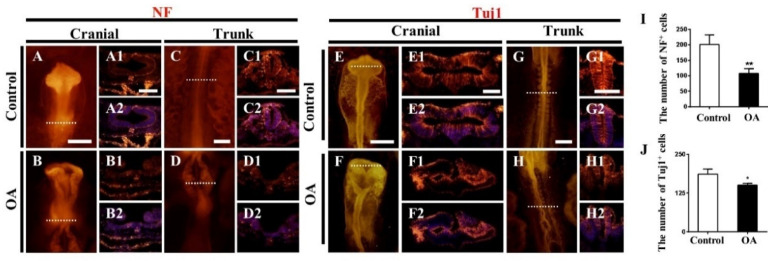
OA exposure inhibits the expression of NF and Tuj1 in neural tubes. (**A**–**D**) and (**E**–**H**) Expressions of NF and Tuj1 on whole-mount embryos, respectively. (**A1**–**D2**) and (**E1**–**H2**) The expression of NF and Tuj1 in the transverse sections at the levels indicated by dotted lines from the whole-mount embryos, respectively. (**I**,**J**) The numbers of NF+ and Tuj1+ cells in the trunk level of chick embryos exposed to OA (n = 3, 3 embryos per replicate). Scale bars = 200 μm in (**A**–**D**) and (**E**–**H**), 100 μm in (**A1**–**D2**) and (**E1**–**H2**). *t*-test, * *p* < 0.05 and ** *p* < 0.01 indicate significant differences between the experimental and control groups.

To investigate the effects of OA on early embryonic neurogenesis, we performed immunofluorescent staining with NF (neurofilament) and Tuj1 (class III β-tubulin) in OA-treated embryos (HH 10). As shown in Figure 3, the expressions of NF (Figure 3A–D2 and I) and Tuj1 (Figure 3E–H2 and J) were significantly reduced after exposure to OA. These indicate that OA exposure inhibit the neuronal differentiation, which might partially contribute to the OA-induced NTDs.

### 2.4. OA Exposure Inhibited Cell Proliferation But Promoted Cell Apoptosis in the Developing Neural Tubes

**Figure 4:**
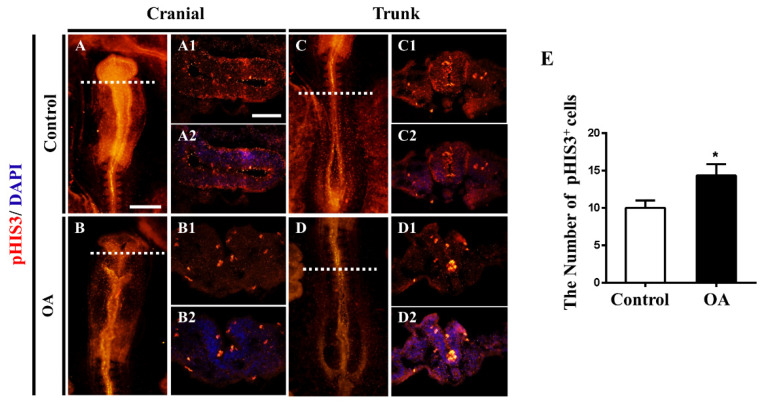
OA exposure promotes cell proliferation in neural tubes. (**A**,**B**) Expression of pHI3 in cranial regions of control and OA-treated groups, respectively. (**C**,**D**) Expression of pHI3 in trunk regions of control and OA-treated groups, respectively. (**A1**,**B1**) and (**C1**,**D1**) Transverse sections at the levels indicated by dotted lines from (**A**,**B**) and (**C**,**D**), respectively. (**A2**,**B2**) and (**C2**,**D2**) Transverse sections from (**A**,**B1**) and (**C1**,**D1**) counterstained with DAPI, respectively. (**E**) A bar chart showing pHIS3+ cell numbers in the transverse sections of control and OA-treated embryos (n = 3, 3 embryos per replicate). Scale bars = 200 μm in (**A**–**D**), 100 μm in (**A1**–**D1**) and (**A2**–**D2**). *t*-test, * *p* < 0.05 indicate significant differences between the experimental and control groups.

To explore whether OA exposure affects the proliferation of neural progenitor cells during neural tube development, we evaluated the cell proliferation of neural progenitors in the developing chick embryos treated with OA using pHIS3 as a cell proliferation marker. As demonstrated in Figure 4A–D2 and E, the number of pHIS3+ cells were significantly increased, suggesting that OA exposure promotes cell proliferation.

**Figure 5:**
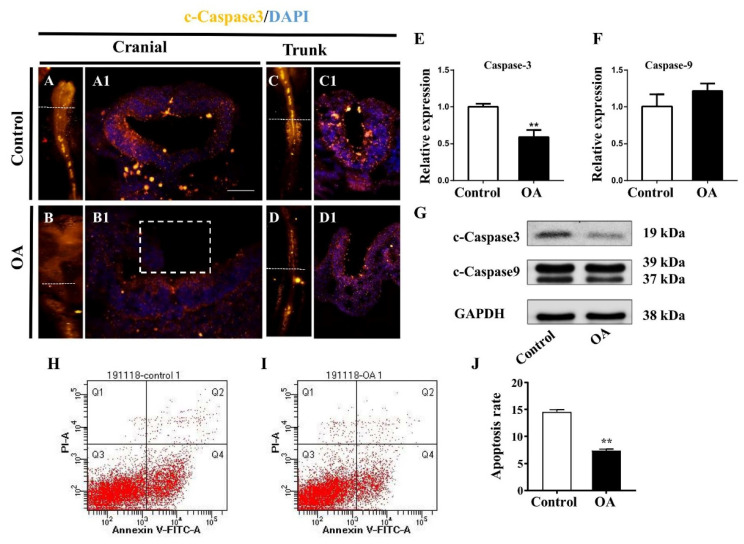
OA exposure inhibits cell apoptosis in neural tubes. (**A,B**) Expression of c-Caspase3 in cranial regions of control and OA-treated groups, respectively. (**C,D**) Expression of c-Caspase3 in trunk regions of control and OA-treated groups, respectively. (**A1**,**B1**) and (**C1**,**D1**) Merge images of transverse sections at the levels indicated by the dotted lines from (**A**,**B**) and (**C**,**D**), respectively. (**E**,**F**) A bar chart showing the transcriptional expression of c-Caspase3 and c-Caspase9 in control and OA-treated embryos (n = 3, 15 embryos per replicate). (**G**) Western blotting data showing the protein expressions of c-Caspase3 and c-Caspase9 in control and OA-treated embryos (n = 3, 16 embryos per replicate). (**H**,**I**) Flow cytometry ‘dot’ plots following staining with propidium iodide and annexin V–FITC of embryo cells in control (**H**) and OA-treated (**I**) groups (n = 3, 15 embryos per replicate). (**J**) The bar chart showing the apoptosis rate based on the flow cytometry analysis (n = 3, 15 embryos per replicate). Scale bars =200 μm in (**A**–**D**), 100 μm in (**A1**–**D1**). *t*-test, * *p* < 0.05 and ** *p* < 0.01 indicate significant differences between the experimental and control groups.

In the same way, we detected changes in the expression of c-Caspase-3 and c-Caspase-9 in the developing neural tubes after being exposed to OA. As shown in Figure 5A–D1, the expression of Caspase-3 significantly decreased both in mRNA (Figure 5E) and protein levels after OA treatment (Figure 5G). However, c-Caspase-9 showed no significant changes in protein and transcriptional levels (Figure 5E,G). Flow cytometry analyses showed that the apoptosis rate was obviously reduced compared with the control counterpart (Figure 5I–J). These results suggest that OA exposure could promote cell proliferation and inhibit cell apoptosis in the neural tubes of early chick embryos.

### 2.5. OA Exposure Induce Oxidative Stress in Early Chick Embryo

**Figure 6:**
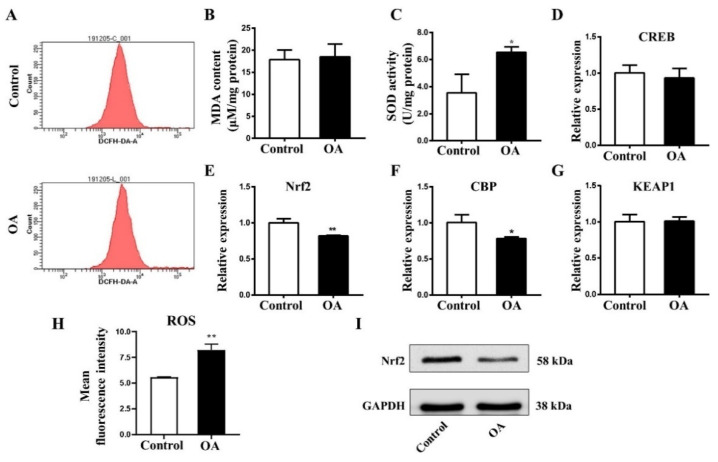
OA exposure induces oxidative stress in chick embryo. (**A**) Flow cytometry results showing content of ROS in control and OA-treated embryos. (**B**) Bar chart showing the content of MDA in control and OA-treated embryos. (**C**) Bar chart showing the activity of SOD after exposed to PBS and OA in control and OA-treated embryos (n = 3, 20 embryos per replicate). (**D**–**G**) The mRNA expressions of CREB (**D**), Nrf2 (**E**), CBP (**F**) and KEAP1 (**G**) in control and OA-treated embryos (n = 3, 15 embryos per replicate). (**H**) Bar chart showing the mean fluorescence intensity (ROS) in control and OA-treated embryos (n = 3, 20 embryos per replicate). (**I**) Western blotting data showing the protein expression of Nrf2 in control and OA-treated embryos, respectively (n = 3, 16 embryos per replicate). *t*-test, * *p* < 0.05 and ** *p* < 0.01 indicate significant differences between the experimental and control groups.

To understand whether oxidative stress is promoted in early chick embryos after OA exposure, flow cytometry was employed to detect ROS content in chick embryos. As in Figure 6, ROS content (Figure 6A,H) and SOD activity (Figure 6B) were evidently increased after OA treatment. However, there were no significant changes in malondialdehyde (MDA) level (Figure 6C). qPCR data demonstrated that *Nrf2* and *CBP* transcripts were markedly down-regulated, while *CREB* and *KEAP1* mRNA levels did not experience any changes (Figure 6D–G). Western blot analyses revealed that Nrf2 expression was distinctly reduced in protein level after OA exposure (Figure 6I). These results indicate that OA exposure induced oxidative stress in early chick embryos and inhibited the Nrf2 signaling pathway.

### 2.6. Effects of OA on BMP/Shh Signaling Molecules

**Figure 7:**
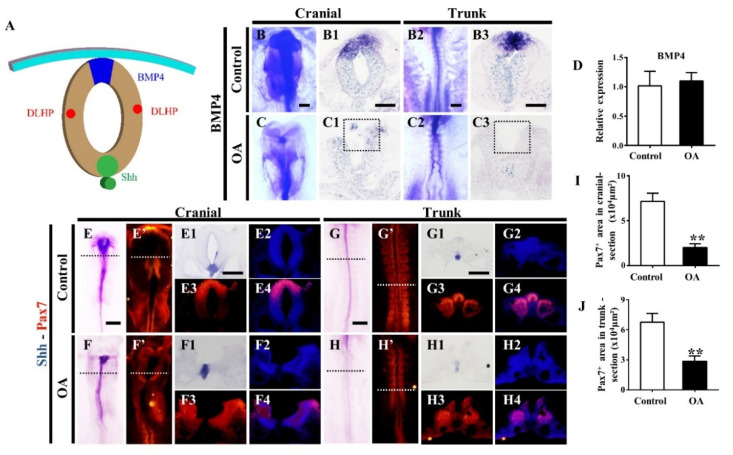
OA exposure represses the expressions of BMP4 and Shh in neural tubes. (**A**) Schematic diagram shows DLHP, BMP4 and Shh expression patterns in the developing neural tubes. (**B**–**C3**) In situ hybridization images of BMP4 in the cranial (**B**,**C**) and trunk (**B2**,**C2**) regions after exposure to OA; the expression of BMP4 in transverse sections at the cranial level (**B1**,**C1**) and trunk level (**B3**,**C3**) after OA treatment. (**D**) qRT-PCR data showing the transcriptional expressions of BMP4 in control and OA-treated embryos (n = 3, 3 embryos per replicate). (**E**–**H4**) Co-expression of Shh-Pax7 at the whole-mount embryos and the corresponding transverse sections after OA treatment (n = 3, 3 embryos per replicate). (**I**,**J**) Pax7+ area in cranial (**I**) and trunk section, respectively (n = 3). Scale bars = 200 μm in **B**–**C** = **B2**–**C2** = **E**–**G** = **E’**–**G’**, 100 μm in **B1**–**C1** = **B3**–**C3** = **E1**–**E4** = **F1**–**F4** = **G1**–**G4**= **H1**–**H4**. *t*-test, ** *p* < 0.01 indicate significant differences between the experimental and control groups.

As presented in Figure 7A, BMP4 and Shh signaling pathways play important roles in regulating the formation of dorsolateral hinge points (DLHPs). The results of *in situ* hybridization demonstrated that the expression of *BMP4* was significantly inhibited both at the cranial and trunk regions after OA exposure compared with the control group (Figure 7B–C3). However, qPCR data showed no significant difference in *BMP4* transcription (Figure 7D). Compared with the control group, the Shh expression in the dorsal neural tubes was obviously reduced after OA treatment (Figure 7E–H4). Accordingly, the expression of Pax7 on the dorsal part of cranial and trunk neural tubes was decreased in the OA-treated embryos (Figure 7E’–H’ and I–J). These results suggest that the formation of NTDs might be related to the inhibition of Pax7 and *BMP4* expression induced by OA exposure.

### 2.7. Transcriptome Analysis and qPCR Validation

Samples were sequenced on the BGISEq-500 platform, and an average of 6.94Gb data were produced for each sample. The raw data were deposited in the NCBI SRA database (https://dataview.ncbi.nlm.nih.gov/object/PRJNA673393?reviewer=hcgkqljjc0sfffpsu7tdla9gem, accessed on 23 March 2021).The ratio of clean reads to raw reads was greater than 93.73%, and the ratio of clean reads Q2 was greater than 97.5%, indicating that the sequencing quality was reliable (Table S1). All the correlation coefficients of biological replications were higher than 0.979, suggesting that the expression patterns of the three replications under the same treatments were highly similar (Figure S1). The average ratio of samples to genome was 89.72% and the ratio of gene set was 78.82% (Table S2).

**Figure 8:**
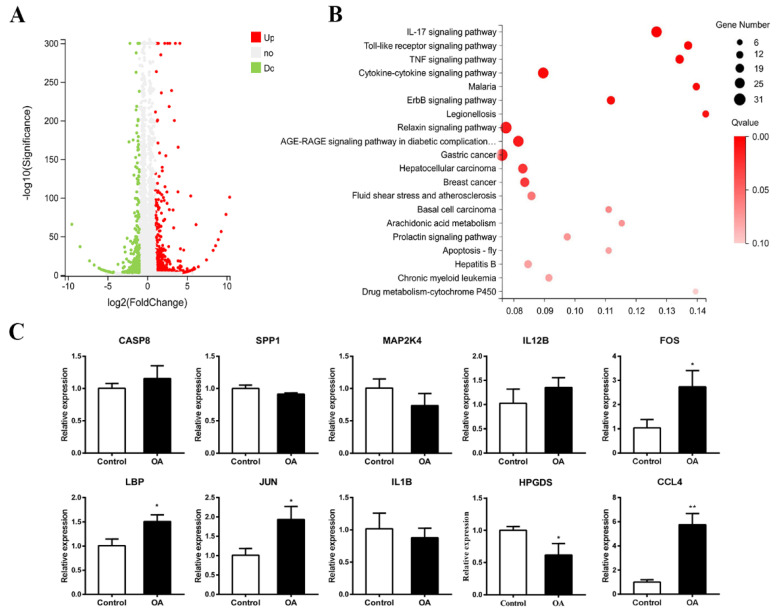
Differential expression gene analyses and qPCR validation. (**A**) Volcano diagram of differential genes. (**B**) KEGG pathway enrichment analysis after OA-treatment. (**C**) qPCR analyses of DEGs on Toll-like receptor signaling pathway after OA exposure (n = 3, 15 embryos per replicate). *t*-test, * *p* < 0.05 and ** *p* < 0.01 indicate significant differences between the experimental and control groups.

Among the 782 DEGs detected, 485 genes were up-regulated (red) and 297 down-regulated (green) (Figure 8A). Based on the GO (Gene Ontology) enrichment analyses, these DEGs are mainly distributed in “biological regulation”, “cellular process”, “metabolic process”, “response to stimulus”, “cell”, “catalytic activity”, “membrane” and “binding” (Figure S2). According to the KEGG pathway category, 45 DEGs were annotated to “cell growth and death”, 146 DEGs were annotated to “signal transduction”, 26 DEGs were annotated to “folding classification and degradation”, 102 DEGs were annotated to the “immune system”, 29 DEGs were annotated to the “nervous system”, and 38 DEGs were annotated to “development” (Figure S3). In the KEGG pathway enrichment analyses, “drug metabolism-cytochrome P450 (CYP450)”, “legionellosis” and “malaria” had the highest proportion of DEGs (about 0.14) (Figure 8B). In addition, the enrichment ratios of the Toll-like receptor signaling pathway, IL-17 signaling pathway and TNF signaling pathway were also close to 0.14, with a high number of DEGs and low Q-values (Figure 8B).

The representative differentially expressed genes induced by OA exposure are summarized in Table 1, which indicated that the multiple key genes involved in the Toll-like receptor signaling pathway such as *LBP* (lipopolysaccharide-binding protein), *JUN* (transcription factor AP-1), *FOS* (proto-oncogene protein c-fos) and *CCL4* (C-C motif chemokine 4), were significantly increased after exposed to OA. In addition, most assayed genes displayed similar expression levels as detected in transcriptome analysis, as demonstrated in Table 2 and Figure 8, which corroborated the transcriptome data.

## 3. Discussion

Several studies have revealed that OA exposure can delay embryonic development and increase embryo mortality of the fish *O. latipes* [18,28], the frog *X. laevis* [17], the longfin yellowtail *Seriola rivoliana* [29], and the chicken *G. gallus* [19]. More importantly, it has been shown that OA can cross the placental barrier, suggesting that OA may cause more harm to fetuses compared to adults since fetus is more vulnerable [20]. As the symptoms of DSP are very similar to gastroenteritis, OA’s neurodevelopmental toxicity is often overlooked by investigators. Hence, it is necessary to evaluate the neurodevelopmental toxicity of OA exposure during pregnancy.

Thus far, OA concentration was set in a range of 5–1000 nM in studies concerning toxicity of OA, so we exposed HH 10 chick embryos to the culture media with 20, 50, 100, 200 and 500 nM of OA. We found that OA exposure could increase the incidences of NTDs and fetal mortality. When the developing chick embryos were exposed to OA at 200 nM and 500 nM, the embryonic mortality rate was about 85% and 65%, respectively. When the embryos were exposed to OA at 100 nM, the embryo malformation rate was at its highest, about 50%. Therefore, we finally chose 100 nM as the experimental concentration and analyzed the abnormal phenotype in both cranial and trunk levels. It is of note that the mortality and deformity rate are significantly reduced when exposed to OA at 500 nM compared to that at 200 nM. This phenomenon may be related to a certain defense threshold, at which point it can activate defense and repair mechanisms, thereby reducing damage [30]. Valdiglesias et al. (2011) [31] and McCarthy et al. (2014) [30] have observed similar results in different cell types. Valdiglesias et al. (2011) [31] found that DNA damage was increased at the lower concentrations (10, 20 and 50 nM), but there was no significant oxidative damage at 1000 nM in SHSY5Y cells. McCarthy et al. (2014) [30] also did not find a classic dose response in the hemolymph and hepatopancreas cells of two bivalves after OA exposure. Conversely, they found a greater increase in DNA fragmentation in the mussel hepatopancreas cells exposed to 1.2 nM than those exposed to 50 nM OA.

To explore the underlying mechanisms of neural tube malformation induced by OA, we first analyzed the neuronal differentiation in the neural tube of chicken embryos. Neurofilament (NF), an intermediate filament protein in the cytoplasm of neurons, is the most abundant component in the cytoskeleton and myelinated axons of mature neurons [32]. The normal expression of NF is closely related to the growth and regeneration of axons and plays an important role in the maintenance of neuronal homeostasis [33]. The abnormal development of neurofilament may lead to a variety of diseases, including ALS (amyotrophic lateral sclerosis), AD (Alzheimer’s disease) and CMT (Charcot–Marie–Tooth) [32]. Tuj1 is a class III member of the β-tubulin protein family. Its expression correlates with the earliest phases of neuronal differentiation. As a marker for the recognition of positive neurons, it has been widely used in many studies since the human brain was found to produce new neurons from neural stem cell pools [34]. The decrease in NF and Tuj1 expressions suggest that OA exposure disrupted the neuronal differentiation and might eventually facilitate the NTDs.

To further understand the causes for the inhibition of neuronal differentiation induced by OA exposure, we used pHIS3, a proliferation marker, to detect cell proliferation in the neural tube. The results showed that the number of neural tube cells in the proliferative state increased, suggesting that OA exposure could promote cell proliferation. Similarly, we also found that OA exposure could inhibit the cell apoptosis in the neural tube. As the specific inhibitors of protein phosphatases PP1 and PP2A, it has been reported that OA could induce cell apoptosis in many cell types [35]. However, several studies have also shown that OA could also block apoptosis through inhibiting PP2A activity [36,37,38]. OA could protect SH-SY5Y cells from 1-methyl-4-phenylpyridinium ion-induced apoptosis [38]. These conflicting outcomes obviously imply the complexity of OA effect on cell apoptosis.

It has been reported that oxidative stress could cause NTDs through suppressing the expressions of related genes [39]. Therefore, we speculate that OA-induced oxidative stress may play an important role in this process. Transcription factor NFE2-related factor (Nrf2) is an important transcription factor, conferring protection against oxidative damage by orchestrating antioxidant and detoxification responses to oxidative stress [40]. After exposure to OA, the increase in ROS content and SOD activity indicate that OA exposure could cause oxidative stress in early chick embryos. The down-regulation of Nrf2 corroborated OA exposure-inhibited Nrf2 signaling pathway, and this in turn aggravated oxidative stress in chick embryos [40]. These results suggest that oxidative stress induced by OA also account for the formation of NTDs.

The formation of dorsolateral hinge points (DHLPs) plays an important role in neural tube closure during neurogenesis [41]. Bone morphogenetic protein (BMP) signaling and Sonic hedgehog (Shh) signaling jointly regulate the formation of DHLP, subsequently affecting the closure neural tube [42]. Neural tube development is highly dependent on the precisely spatiotemporal regulation of bone morphogenetic protein 4 (BMP4), paired box 7 (Pax7) and Sonic hedgehog (Shh) genes in the dorsal side of the tube [43]. After exposure to OA, the inhibition of *BMP4*and *Shh* expression in the dorsal neural tube suggests that OA exposure could also affect the formation of DHLP, and then disturb the subsequent folding process, and ultimately lead to the incomplete closure of the neural tube.

To understand the underlying molecular mechanisms by which OA exposure induces neural tube defects, we performed transcriptomic sequencing on the early chick embryos. A total of 782 differentially expressed genes were obtained with 485 up-regulated genes and 297 down-regulated ones. These DEGs were mainly enriched in cytokine–cytokine receptor interaction, Toll-like receptor signaling pathway, IL-17 signaling pathway and TNF signaling pathway. To further assess whether OA exposure can activate the Toll-like receptor signaling pathway in the early chicken embryos, we observed the expression of some genes related to the Toll-like receptor signaling pathway by using qPCR. As our expected, the expressions of *LBP*, *JUN*, *FOS* and *CCL4* in the Toll-like receptor signaling pathway were significantly increased after exposure to OA, which is consistent with the results of transcriptome sequencing. The slight discrepancy between transcriptome analysis and qPCR might be due to the diversity in the statistical processing of data [44].

Toll-like receptors (TLRs) play a crucial role in the innate immune system by recognizing pathogen-associated molecular patterns derived from various microbes [45]. FOS, the immediate early transcription factor of neurons, is closely connected with neuronal programmed cell death [46]. c-Fos is a member of FOS family proteins (Fra-1, Fra-2, FosB), which can heterodimerize with members of JUN family (c-Jun, JunB, JunD) to form transcription factor activator protein 1 (AP-1) [47]. AP-1 transcription factor complexes can affect cell proliferation and differentiation via regulating gene expression in response to positive and negative stimuli [48]. Shaulian and Karin (2001) [49] found that cell proliferation and cell cycle were inhibited in mouse fibroblasts and erythroleukemia cell lines when the expressions of *FOS* and *JUN* were suppressed by antisense RNA. Kovary and Bravo (1991) [50] reported that the microinjection of anti-Fos and anti-Jun antibodies efficiently prevented serum-stimulated or asynchronously growing cells from entering the S phase. In our study, *FOS* and *JUN* expressions were significantly up-regulated after OA exposure, which could explain why the numbers of apoptotic cells in the neural tube decreased while proliferating cells increased in the OA-treated embryos.

Chemokine CC (motif) ligand 4 (*CCL4*), also named macrophage inflammatory protein-1β (*MIP-1β*), is essential for chemotaxis of macrophages, natural killer cells, and lymphocytes [51]. *CCL4* is secreted from glial and astrocytes, and involved in the progression of various brain diseases, including Alzheimer’s disease, multiple sclerosis, and ischemic brain disease, though its function in the brain remains unclear [52]. Several reports have shown that the recombinant *CCL4* can attenuate the toxicity of methylmercury (MeHg) to primary neurons in mice, while *CCL4* knockdown in C17.2 cells results in higher MeHg sensitivity compared with control cells [52]. The up-regulation of *CCL4* in the OA-exposed embryo might be a protective response of embryos to OA exposure-induced toxicity.

As a key participant in the inflammatory response to infection, LBP is a type I acute phase response protein produced by a variety of cell types, which can enhance the recognition of endotoxins and pathogens by the immune system [53]. Studies have manifested that LBP plays an important protective role in alcoholic-induced liver injury [54]. Recently, Pretorius et al. (2018) [55] found that LBP could reverse the presence or induction of fibrin amyloid in Parkinson’s disease. Based on the significant reduction in egg-laying in *Biomphalaria glabrata* after the silencing of LBP/BPI1 expression, Baron et al. (2013) [56] consider that LBP may be involved in prenatal immune protection of offspring. The increased expression of *LBP* after OA exposure might also be a protective response to OA exposure-induced toxicity.

The chicken embryo has been used as a standard animal model for embryonic development, especially embryonic neural development, for nearly a century [57]. An unexpected result of the linkage mapping suggests that the chicken genome is more closely related to the human genome than the mouse genome [58]. The early chick embryo model corresponds to the first month of mammalian embryonic development [59]. The development of neuron and spinal cords in chick embryo is very similar to the development of human embryos [60]. Therefore, it is feasible to use chicken embryos to study the development of human embryos in the early stage of embryo development, which can reveal the development of human embryos to a great extent. Our finding may provide some reference for the toxicity of OA to human embryo development.

## 4. Materials and Methods

### 4.1. Chemicals

Okadaic acid (Purity ≥ 95% by HPLC, Zaoyan, Taoyuan, China) was dissolved in dimethyl sulfoxide (DMSO) at the stock concentration of 1 mM. The stock concentration of OA was then diluted with phosphate-buffered saline (PBS) to a concentration of 100 nM.

### 4.2. Ethical Statement

The animal protocols used in this work were evaluated and approved by the Laboratory Animal Ethics Committee of Jinan University (IACUC-20181126-02, 2018-11-26).

### 4.3. Chick Embryos

Fertilized chicken eggs were purchased from an avian farm in South China Agricultural University in Guangzhou, China. For the early stage of chick embryos, early chick (EC) culture was employed as described previously [61]. The agar–albumen medium was prepared as described in our previous study [19]. The HH1 chick embryos were incubated with PBS (control) or the culture media containing different concentrations of OA in an incubator (37 °C and 70% humidity) (Boxun, Shanghai, China) until the embryos developed to the desired stage (HH10). For the late stage of chick embryos, the eggs that were pre-incubated for 1.5 days were administrated with the same volume of PBS or OA through a pre-windowed small hole, and then incubated in an incubator (37 °C and 70% humidity) for a further 3 days. The holes were sealed with UV-irradiated transparent tape to avoid dehydration and contamination.

### 4.4. Immunofluorescent Staining

The HH 10 chick embryos were fixed in 4% paraformaldehyde (PFA) overnight at 4 °C. The primary antibodies, including (NF, 1:200, Life Technologies, Carlsbad, CA, USA), Tuj1 (1:200, Neuromics, Edina, MN, USA), pHIS3 (1:400, Santa Cruz, Dallas, TX, USA) and c-Caspase3 (1:400, Cell Signaling Technology, Boston, MA, USA), were employed in the immunofluorescent staining of whole-mount embryos. Pax7 (1:400) was obtained from the Developmental Studies Hybridoma Bank (DSHB), created by the NICHD of the NIH and maintained at the University of Iowa, Department of Biology, Iowa City, IA 52242. Briefly, the fixed HH 10 chick embryos were incubated with the primary antibody at 4 °C overnight on a shaker and then washed carefully in PBST (0.1% tween-20). Next, the embryos were washed with PBT for 5 min, then blocked in blocking buffer for 6 h. Subsequently, they were incubated with a related Alexa Fluor^®^ 488 or 555 labelled secondary antibodies (1:1000, Invitrogen, Carlsbad, CA, USA) at 4 °C overnight on a shaker. Finally, all the chick embryos were counterstained with DAPI (1:1000, Invitrogen, Carlsbad, CA, USA) at room temperature for 40 min. After being photographed, the stained embryos were embedded in a solution of 7.5% gelatin–15% sucrose (w/v) and stored at −80 °C. Whereafter, the embedded embryo was sectioned at a thickness of 12 μm using a freezing microtome (Leica CM1900, Nussloch, Germany).

### 4.5. In Situ Hybridization

*In situ* hybridization of whole-mount chick embryo was carried out according to the method described previously [62]. Briefly, HH 10 chick embryos were fixed in 4% paraformaldehyde overnight at 4 °C, washed twice with PTW (0.1% tween-20 dissolved in PBS) for 5 min each time, and in a graded series of methanol (25%, 50%, 75%, 100%) for 5 min, respectively. After being rehydrated in methanol (75%, 50%, 25%) and PTW for 5 min, respectively, the embryos were incubated in hybridization buffer for 5 h. Subsequently, antisense probes were added to the cultures, and the embryo were incubated overnight at 65 °C. Digoxigenin-labeled antisense probes were generated to specifically detect mRNA levels of bone morphogenetic protein 4 (*BMP4*) and Sonic hedgehog (*Shh*). The primer sequences used in probes are summarized in Table 3. After hybridization, the embryos were washed by using post-hybridization buffer and TBST (0.1% tween -20 dissolved in TBS) twice for 30 min at 65 °C, respectively. After being blocked with a blocking reagent (Roche, Basel, Switzerland) for 5 h, the embryos were incubated with anti-DIG (digoxigenin) antibody (1:1000, Roche, Basel, Switzerland) overnight at 4 °C on a shaker. Finally, the embryos were incubated in BCIP/NBT chromogen solution (Sigma, Santa Clara, CA, USA) at room temperature for staining. The stained embryos were pictured and sectioned at a thickness of 16 μm using a freezing microtome (Leica CM1900, Nussloch, Germany). Image-Pro Plus 7.0 (IPP 7.0) was employed to calculate the area of the target region (labelled with probes).

### 4.6. Fluorescent Microscopy

After immunofluorescent staining or in situ hybridization staining, the stained embryos and the regions of interest were pictured using a stereo-fluorescence microscope and processed with Image-Pro Plus 7.0 (IPP 7.0). The sliced embryos were pictured by using an Olympus IX51epi-fluorescent microscope (Olympus, Tokyo, Japan), and the obtained pictures were analyzed with a Leica CW4000 FISH software (Leica Microsystems, Nussloch, Germany).

### 4.7. RNA Isolation and Quantitative Real-Time PCR Analysis

Total RNA was extracted from the HH10 chick embryos using a total RNA kit (R6834-01, Omega, Norcross, GA, USA) based on the manufacturer’s instructions. Some of the RNA isolated was subjected to high-throughput sequencing, while others were used to reverse transcription. Agarose gel electrophoresis and NanoDrop 2000 (Thermo Scientific, Waltham, MA, USA) were employed to evaluate the integrity, concentration, and purity of RNA for reverse transcription, respectively. First-strand cDNA was generated from 1 μg of total RNA by using a HiScript^®^ II Q RT SuperMix for qPCR (+gDNA wiper) (Vazyme, Nanjing, China). The integrity, concentration, and purity of RNA for high-throughput sequencing were determined by an Agilent 2100 Bioanalyzer and RNA Nano 6000 assay kit (Agilent Technologies, Palo Alto, CA, USA).

Specific primers employed in this study were designed by Primer 5.0. Reference genes were screened using geNorm, NormFinder and BestKepper. Among the six candidate genes, including glyceraldehyde 3-phosphate dehydrogenase (*GAPDH*), ubiquitin A-52 (*UBA52*), cyclophilin-A, succinate dehydrogenase complex subunit A (*SDHA*), ribosomal protein S15e (*RPS15*) and ribosomal protein L30 (*RPL30*), *RPS15* and *RPL30* exhibited the most stable expression. Therefore, the two genes were employed as reference genes to normalize the expression of target genes. The primer characters used in RT-qPCR are summarized in Table 4. The PCR reaction system and procedure were performed as described in our previous paper [63].

The comparative Cq method was employed to analyze the relative expression of target genes as described by Hellemans et al. (2007) [64], in which multiple reference genes and inter-run calibration algorithms were considered. Standard curves were generated to check the efficiency of PCR amplification [65]. Amplification efficiency for each reaction should vary from 0.900 to 1.110, while correlation coefficients range between 0.990 and 0.999.

### 4.8. RNA-seq Assay

The sequencing was conducted in BGI-Shenzhen (Shenzhen, China). Total RNA was qualified and quantified using a NanoDrop and Agilent 2100 bioanalyzer (Thermo Fisher Scientific, Waltham, MA, USA). Oligo (dt)-attached magnetic beads were used to purify mRNA. Purified mRNA was randomly fragmented into small pieces, and sequencing libraries were established using a MGIEasy RNA-seq library prep kit (BGI-Shenzhen, China) based on the manufacturer’s instructions. The library quality was assessed on the Agilent Bioanalyzer 2100 system. The final library was then sequenced on the BGISEQ-500 platform (BGI-Shenzhen, China) at paired-end mode (PE150).

Trimmomatic (Version 0.36) was employed to trim adapters and low-quality bases, and Q20 was chosen for quality trimming [66]. Bowtie2 (Version 2.2.5) was applied to align the clean reads to the reference coding gene set, then the expression level of genes was calculated by RSEM (v1.2.12) [67]. Differential expressed genes (DEGs) analyses were performed using the DESeq2 (v1.4.5) [68] with |Fold Change | ≥ 2 and Q-values ≤ 0.001. Blast2GO [69] (Release 5.2.4, 10. 2018) and the Kyoto Encyclopedia of Genes and Genomes (KEGG, https://www.kegg.jp/, release 89.1, accessed on 1 February 2019) enrichment analyses of annotated different expressed genes were performed with Phyper based on hypergeometric test. The significant levels of terms and pathways were corrected by Q-values with a rigorous threshold (Q value ≤ 0.05) by Bonferroni [70].

### 4.9. Western Blot

The total protein concentration was measured by using a BCA Protein Assay Kit (Beyotime, China) according to the manufacturer’s instructions. The samples containing equal amounts of proteins were separated by 12% SDS-PAGE and transferred to PVDF membranes (Millipore, Bedford, OH, USA). The membranes were blocked with 5% Difco™ skim milk (BD, Franklin Lakes, NJ, USA), and then incubated with primary and secondary antibodies. All primary and secondary anti-bodies used were diluted to 1:1000 and 1:3000 in 5% skim milk, respectively. The protein bands were visualized with an ECL substrate kit (BIO-RAD, Hercules, CA, USA). The antibody-stripped membrane was then blocked again and re-incubated with other antibodies.

### 4.10. Detection of MDA Content and SOD Activity

Thirty HH10 chick embryos were harvested from each experimental group. The ten embryos within the same treatment were pooled together as one sample, and each group contained three pooled samples. The malondialdehyde (MDA) levels and superoxide dismutase (SOD) activities were detected in tree homogenized samples isolated from control or OA-treated groups according to the manufacturer’s instructions. The MDA content was measured by using a lipid peroxidation MDA assay kit (Beyotime, Shanghai, China). SOD activity was determined by using a total superoxide dismutase assay kit with WST-8 (Beyotime, Shanghai, China). A microplate reader (Tecan Sunrise, Männedorf, Switzerland) was available for absorbance detection in the experiments.

### 4.11. Flow Cytometry Analysis

The HH10 chick embryos were harvested in a cell culture dish on a clean bench. After being rinsed with sterilized PBS (phosphate buffer saline), the tissue was transferred to a sterile centrifuge tube. To the centrifuge tube, trypsin (0.25%) was added, and the tissue was blown to homogenate with a pipette. Cell culture medium (Gibco, GrandIsland, NY, USA) was introduced to terminate trypsin digestion. Finally, the mixture was filtered with a 200-mesh sterile cell filter sieve (Jingan, Shanghai, China), and the filtered cell suspension was centrifuged at 1600× *g* for 2 min, and liquid was discarded.

For the analysis of apoptosis, we re-suspended cells in 100 µL of binding buffer (BD, USA), then added 2.5 µL of annexin V-FITC and propidium iodide (PI) to the cell suspension. Thereafter, another 200 µL of the binding buffer were added to the mixture. After being incubated for 15 min at room temperature in the dark, the cell suspension was transferred to the upper sample tube. For the detection of reactive oxygen species (ROS) content, we re-suspended cells in 200 µL of dichlorofluorescein diacetate (DCFH-DA) reagent and incubated at room temperature for 15 min in the dark. After being washed with sterile PBS, the cell suspension was centrifuged at 1600× *g* for 5 min. Finally, we re-suspended cells in 300 µL of PBS and transferred it to the upper sample tube for ROS detection. Flow cytometry analysis was performed on the FACSCanto (BD, USA) system.

### 4.12. Statistical Analyses

Statistical analyses were carried out by using GraphPad Prism 7 software (GraphPad, San Diego, CA, USA). All data are presented as mean ± SD. After testing for homogeneity of variance, Student’s t-test was employed to check the differences between two groups. Multiple group comparisons of the means were performed by one way ANOVA. * *p* < 0.05 was considered to be statistically significant and ** *p* < 0.01 was considered to be highly statistically significant.

## 5. Conclusions

OA exposure can cause neural tube defects in early chick embryos and increase the incidences of embryo mortality and malformation. OA exposure can alter the expressions of BMP4 and Shh, affect the formation of DLHP, and ultimately hinder the closure of the neural tube. OA exposure can cause oxidative stress in early chick embryos, which may be subsequently responsible for the formation of NTDs. OA exposure can affect cell proliferation and apoptosis through the Toll-like receptor signaling pathway. Our findings provide a new basis for the comprehensive evaluation of the neural developmental toxicity of OA during pregnancy. However, we should keep in mind that neural tube closure is a complex and precise process concerning the regulation of multiple signaling pathways. There is no doubt that much more precise works are required to explore the molecular mechanisms of neural tube defects induced by OA exposure in the future.

## Figures and Tables

**Table 1:** Representative differentially expressed genes in the early chicken embryos after exposure to OA.

ID	KO	Name	Product	Log_2_(FC)	Q-Value
107056355	K10030	IL8	interleukin 8	−1.74	5.08 × 10^−85^
107056614	K04398	CASP8	caspase 8	1.47	1.13 × 10^−62^
107057160	K04430	MAP2K4	mitogen-activated protein kinase kinase 4	10.38	1.4 × 10^−101^
395196	K04519	IL1B	interleukin 1 beta	3.86	1.76 × 10^−8^
395210	K06250	SPP1	secreted phosphoprotein 1	1.03	0
395468	K12964	CCL4	chemokine CC motif ligand 4	3.54	0
395872	K10030	IL8L1	interleukin 8-like 1	7.46	2.33 × 10^−20^
396330	K09447	IRF7	interferon regulatory factor 7	1.49	1.71 × 10^−128^
396512	K04379	FOS	proto-oncogene protein c-fos	2.19	0
404671	K05425	IL12B	interleukin 12B	1.77	7.7 × 10^−20^
416548	K17783	ERV1	mitochondrial FAD-linked sulfhydryl oxidase	−1.01	2.29 × 10^−19^
424673	K04448	JUN	transcription factor AP-1	1.27	0
771461	K05399	LBP	lipopolysaccharide-binding protein	1.37	2.1 × 10^−10^
395863	K04097	HPGDS	hematopoietic prostaglandin D synthase	1.75	3.71 × 10^−25^

**Table 2:** Changes of partial differences in gene expression.

Genes	Transcriptome Results Log_2_(FC)	qPCR Results
CASP8	1.47	1.16
MAP2K4	10.38	-
IL1B	3.86	-
SPP1	1.03	-
CCL4	3.54	-
FOS	2.19	2.73
IL12B	1.77	-
JUN	1.27	1.94
LBP	1.37	1.51
HPGDS	1.75	−0.62

**Table 3:** Probe primer sequences for in situ hybridization in this study.

Genes	Primer Sequence 5′-3′	Products Size (bp)
BMP4	F: TTATAAAAGCTTGCGGCCGCAGAATATATGTTTGGCTGCGAAGGC R: GCTCTAGAAATTAACCCTCACTAAAGGGCGTGGTTGGTGGAGTTGAG	860
Shh	F: CCATCACTCCGAGGAATCGC R: AATTAACCCTCACTAAAGGGAGACCCAGCACATAGACACGTTG	525

**Table 4:** Primer sequences used in this study.

**Genes**	**Primer Sequence 5′-3′**	**Products Size (bp)**
RPL3	F: CTGGTGATGAAAAGCGGTAA R: CAAAGCAGGACAGTTGTTGG	108
RPS15	F: TTCCGCAAGTTCACCTACAG	165
	R: CAAAGCAGGACAGTTGTTGG	
CASP8	F: TGGGAAAGTGGACAAGAGCC R: CATCTCTCCTTCACCAAGTAAGT	73
MAP2K4	F: GCATGCAGGGTAAACGCAAA R: AACCTTGCCGTGGACTTGAA	70
SPP1	F: GAGCGTAGAGAACGACAGCC R: CTCTAGCGTCTGGTTGCTGG	139
CCL4	F: AGCCTCCTCTGCCCCAG R: TCGCGCTCCTTCTTTGTGAT	153
FOS	F: GCCGACATGATGTACCAGGG R: GACGGGTAGTAGGTGAGGCT	101
IL12B	F: CACCAGCCGACTGAGATGTT R: GAGGTGGGTCTGGCTTTATGAT	103
LBP	F: AAGGTTTGTGACAGCGTGGT R: ACGTTTGCTTCTGGCAAGGT	77
HPGDS	F: GCCATTCCAACTGCATTCCC R: TTTTCTCCCTCTGCGAACCC	84
CREB	F: AATGGATCTCTTGGGGCAGC R: ACCTGCCATTCCCATTTTTGT	186
CBP	F: CCTCAACCACATGACGCACT R: GGCCGTCTTGAAGCTCATCTC	111
Nrf2	F: GGCCGTCTTGAAGCTCATCTC R: TGCCTCTCCTGCGTATATCTCG	175
Caspase3	F: CCACCGAGATACCGGACTGT R: GGAATGAGGACGAGCCAGAC	173
Caspase9	F: GGAATGAGGACGAGCCAGAC R: GTACCACGAGCCACTCACCTT	119
KEAP1	F: CTTCGCTGAGGTCTCCAAG R: CAGTCGTACTGCACCCAGTT	142
IL1B	F: GGAGAGCAGCAGCCTCAG R: AGCCCTCCCATCCTTACCTT	79
KEAP1	F: ACTTCGCTGAGGTCTCCAAG	142
	R: CAGTCGTACTGCACCCAGTT	
JUN	F: CCTCCCCTGTCCCCTATTGA	99
	R: CCTTTTCCGGCATTTGGACG	

## Data Availability

The datasets presented in the current study are available from the corresponding authors on reasonable request. The transcriptome datasets analyzed during the current study are available in the NCBI Sequence Read Archive (SRA) (http://www.ncbi.nlm.nih.gov/sra/, 30 October 2020) under PRJNA673393.

## Supplementary Materials

The following are available online at https://www.mdpi.com/article/10.3390/md19060322/s1, Figure S1: Classification map of GO annotation after OA treatment, Figure S2: Differential gene KEGG Pathway classification after exposed to OA, Figure S3: Correlation between different biological replicates, Table S1: Reads quality statistics, Table S2: Reference genome alignment statistics.

## References

[1] Valdiglesias V., Prego-Faraldo M., Pásaro E., Méndez J., Laffon B. (2013). Okadaic acid: More than a diarrheic toxin. Mar. Drugs.

[2] Reguera B., Riobó P., Rodríguez F., Díaz P., Pizarro G., Paz B., Franco J., Blanco J. (2014). Dinophysis toxins: Causative organisms, distribution and fate in shellfish. Mar. Drugs.

[3] James K.J., Carey B., O’Halloran J., van Pelt F.N.A.M., Škrabáková Z. (2010). Shellfish toxicity: Human health implications of marine algal toxins. Epidemiol. Infect..

[4] Vale C., Botana L.M. (2008). Marine toxins and the cytoskeleton: Okadaic acid and dinophysistoxins. FEBS J..

[5] Bialojan C., Takai A. (1988). Inhibitory effect of a marine-sponge toxin, okadaic acid, on protein phosphatases. Specificity and kinetics. Biochem. J..

[6] Holmes F.B., Luu H.A., Carrier F., Schmitz F.J. (1990). Inhibition of protein phosphatases-1 and -2A with acanthifolicin: Comparison with diarrhetic shellfish toxins and identification of a region on okadaic acid important for phosphatase inhibition. FEBS Lett..

[7] Valdiglesias V., Fernández-Tajes J., Costa C., Méndez J., Pásaro E., Laffon B. (2012). Alterations in Metabolism-Related Genes Induced in SHSY5Y Cells by Okadaic Acid Exposure. J. Toxicol. Environ. Health Part A.

[8] Fu L.L., Zhao X.Y., Ji L.D., Xu J. (2019). Okadaic acid (OA): Toxicity, detection and detoxification. Toxicon.

[9] Nuydens R., De Jong M., Van Den Kieboom G., Heers C., Dispersyn G., Cornelissen F., Nuyens R., Borgers M., Geerts H. (1998). Okadaic acid-induced apoptosis in neuronal cells: Evidence for an abortive mitotic attempt. J. Neurochem..

[10] Valdiglesias V., Laffon B., Pásaro E., Méndez J. (2011). Okadaic acid induces morphological changes, apoptosis and cell cycle alterations in different human cell types. J. Environ. Monit..

[11] Wang R., Li J., Zhao Y., Xing Y., Xue X., Zhang J. (2017). Effects of okadaic acid combined with cisplatin on the proliferation and apoptosis of human lung adenocarcinoma A549 cells. Int. J. Clin. Exp. Med..

[12] Dietrich J., Schindler M., Lampen A., Braeuning A., Hessel-Pras S. (2020). Comparison of long-term versus short-term effects of okadaic acid on the apoptotic status of human HepaRG cells. Chem.-Biol. Interact..

[13] Çakır M., Tekin S., Doğanyiğit Z., Erden Y., Soytürk M., Çiğremiş Y., Sandal S. (2019). Cannabinoid type 2 receptor agonist JWH-133, attenuates Okadaic acid induced spatial memory impairment and neurodegeneration in rats. Life Sci..

[14] Chighladze M., Beselia G., Burjanadze M., Dashniani M. (2019). Recognition memory impairment and neuronal degeneration induced by intracerebroventricular or intrahippocampal administration of okadaic acid. Eur. Neuropsychopharmacol..

[15] Kamat P.K., Rai S., Nath C. (2013). Okadaic acid induced neurotoxicity: An emerging tool to study Alzheimer’s disease pathology. Neurotoxicology.

[16] Koehler D., Williams F.E. (2018). Utilizing zebrafish and okadaic acid to study Alzheimer’s disease. Neural Regen. Res..

[17] Casarini L., Franchini A., Malagoli D., Ottaviani E. (2007). Evaluation of the effects of the marine toxin okadaic acid by using FETAX assay. Toxicol. Lett..

[18] Escoffier N., Gaudin J., Mezhoud K., Huet H., Chateau-Joubert S., Turquet J., Crespeau F., Edery M. (2007). Toxicity to medaka fish embryo development of okadaic acid and crude extracts of *Prorocentrum* dinoflagellates. Toxicon.

[19] Jiao Y.H., Liu M., Wang G., Li H.Y., Liu J.S., Yang X.X., Yang W.D. (2019). EMT is the major target for okadaic acid-suppressed the development of neural crest cells in chick embryo. Ecotoxicol. Environ. Saf..

[20] Matias W., Creppy E. (1996). Transplacental passage of [3H]-okadaic acid in pregnant mice measured by radioactivity and high-performance liquid chromatography. Hum. Exp. Toxicol..

[21] Colas J.F., Schoenwolf G.C. (2001). Towards a cellular and molecular understanding of neurulation. Dev. Dyn..

[22] Copp A.J., Greene N.D., Murdoch J.N. (2003). The genetic basis of mammalian neurulation. Nat. Rev. Genet..

[23] Christianson A., Howson C.P., Modell B. (2005). March of Dimes: Global Report on Birth Defects: The Hidden Toll of Dying and Disabled Children.

[24] Manning S.M., Jennings R., Madsen J.R. (2000). Pathophysiology, prevention, and potential treatment of neural tube defects. Ment. Retard. Dev. Disabil. Res. Rev..

[25] Padmanabhan R. (2006). Etiology, pathogenesis and prevention of neural tube defects. Congenit. Anom..

[26] Faez T., Skachkov I., Versluis M., Kooiman K., de Jong N. (2012). In vivo characterization of ultrasound contrast agents: Microbubble spectroscopy in a chicken embryo. Ultrasound Med. Biol..

[27] Lokman N.A., Elder A.S., Ricciardelli C., Oehler M.K. (2012). Chick chorioallantoic membrane (CAM) assay as an in vivo model to study the effect of newly identified molecules on ovarian cancer invasion and metastasis. Int. J. Mol. Sci..

[28] Figueroa D., Signore A., Araneda O., Contreras H.R., Concha M., García C. (2020). Toxicity and differential oxidative stress effects on zebrafish larvae following exposure to toxins from the okadaic acid group. J. Toxicol. Environ. Health Part A.

[29] Le Du J., Tovar-Ramírez D., Núñez-Vázquez E. (2017). Embryotoxic effects of dissolved okadaic acid on the development of Longfin yellowtail *Seriola rivoliana*. Aquat. Toxicol..

[30] McCarthy M., O’Halloran J., O’Brien N.M., van Pelt F.F. (2014). Does the marine biotoxin okadaic acid cause DNA fragmentation in the blue mussel and the pacific oyster?. Mar. Environ. Res..

[31] Valdiglesias V., Laffon B., Pásaro E., Cemeli E., Anderson D., Méndez J. (2011). Induction of oxidative DNA damage by the marine toxin okadaic acid depends on human cell type. Toxicon.

[32] Julien J.P. (1999). Neurofilament functions in health and disease. Curr. Opin. Neurobiol..

[33] Wang H., Wu M., Zhan C., Ma E., Yang M., Yang X., Li Y. (2012). Neurofilament proteins in axonal regeneration and neurodegenerative diseases. Neural Regen. Res..

[34] Jouhilahti E.M., Peltonen S., Peltonen J. (2008). Class III β-tubulin is a component of the mitotic spindle in multiple cell types. J. Histochem. Cytochem..

[35] Lago J., Santaclara F., Vieites J.M., Cabado A.G. (2005). Collapse of mitochondrial membrane potential and caspases activation are early events in okadaic acid-treated Caco-2 cells. Toxicon.

[36] Morana S.J., Wolf C.M., Li J., Reynolds J.E., Brown M.K., Eastman A. (1996). The involvement of protein phosphatases in the activation of ICE/CED-3 protease, intracellular acidification, DNA digestion, and apoptosis. J. Biol. Chem..

[37] Härmälä-Braskén A.S., Mikhailov A., Söderström T.S., Meinander A., Holmström T.H., Damuni Z., Eriksson J.E. (2003). Type-2A protein phosphatase activity is required to maintain death receptor responsiveness. Oncogene.

[38] Ahn K.H., Kim Y.S., Kim S.Y., Huh Y., Park C., Jeong J.W. (2009). Okadaic acid protects human neuroblastoma SH-SY5Y cells from 1-methyl-4-phenylpyridinium ion-induced apoptosis. Neurosci. Lett..

[39] Chang T., Horal M., Jain S., Wang F., Patel R., Loeken M. (2003). Oxidant regulation of gene expression and neural tube development: Insights gained from diabetic pregnancy on molecular causes of neural tube defects. Diabetologia.

[40] Kensler T.W., Wakabayashi N., Biswal S. (2007). Cell survival responses to environmental stresses via the Keap1-Nrf2-ARE pathway. Annu. Rev. Pharmacol. Toxicol..

[41] Yamaguchi Y., Miura M. (2013). How to form and close the brain: Insight into the mechanism of cranial neural tube closure in mammals. Cell. Mol. Life Sci..

[42] Mahalik S.K., Vaze D., Lyngdoh T.S., Tewari M.K., Narasimhan K.L. (2011). Embryogenesis of triple neural tube defects: Sonic hedgehog—A key?. J. Clin. Pediatr. Surg..

[43] Jin Y.M., Wang G., Zhang N., Wei Y.F., Li S., Chen Y.P., Chuai M., Lee H.S.S., Hocher B., Yang X. (2015). Changes in the osmolarity of the embryonic microenvironment induce neural tube defects. Mol. Reprod. Dev..

[44] Dergunova L.V., Filippenkov I.B., Stavchansky V.V., Denisova A.E., Yuzhakov V.V., Mozerov S.A., Gubsky L.V., Limborska S.A. (2018). Genome-wide transcriptome analysis using RNA-Seq reveals a large number of differentially expressed genes in a transient MCAO rat model. BMC Genom..

[45] Kawasaki T., Kawai T. (2014). Toll-like receptor signaling pathways. Front. Immunol..

[46] Curran T., Morgan J.I. (1995). Fos: An immediate-early transcription factor in neurons. J. Neurobiol..

[47] Velazquez F.N., Caputto B.L., Boussin F.D. (2015). c-Fos importance for brain development. Aging.

[48] Van Dam H., Castellazzi M. (2001). Distinct roles of Jun: Fos and Jun: ATF dimers in oncogenesis. Oncogene.

[49] Shaulian E., Karin M. (2001). AP-1 in cell proliferation and survival. Oncogene.

[50] Kovary K., Bravo R. (1991). The jun and fos protein families are both required for cell cycle progression in fibroblasts. Mol. Cell. Biol..

[51] Hsu Y.J., Hou C.Y., Lin S.J., Kuo W.C., Lin H.T., Lin J.H.Y. (2013). The biofunction of orange-spotted grouper (*Epinephelus coioides*) CC chemokine ligand 4 (CCL4) in innate and adaptive immunity. Fish. Shellfish. Immunol..

[52] Takahashi T., Kim M.S., Iwai-Shimada M., Fujimura M., Toyama T., Naganuma A., Hwang G.W. (2018). Chemokine CCL4 induced in mouse brain has a protective role against methylmercury toxicity. Toxics.

[53] Regueiro V., Campos M., Morey P., Sauleda J., Agustí A., Garmendia J., Bengoechea J. (2009). Lipopolysaccharide-binding protein and CD14 are increased in the bronchoalveolar lavage fluid of smokers. Eur. Respir. J..

[54] Uesugi T., Froh M., Arteel G.E., Bradford B.U., Wheeler M.D., Gäbele E., Isayama F., Thurman R.G. (2002). Role of lipopolysaccharide-binding protein in early alcohol-induced liver injury in mice. J. Immunol..

[55] Pretorius E., Page M.J., Mbotwe S., Kell D.B. (2018). Lipopolysaccharide-binding protein (LBP) can reverse the amyloid state of fibrin seen or induced in Parkinson’s disease. PLoS ONE.

[56] Baron O.L., Van West P., Industri B., Ponchet M., Dubreuil G., Gourbal B., Reichhart J.M., Coustau C. (2013). Parental transfer of the antimicrobial protein LBP/BPI protects *Biomphalaria glabrata* eggs against oomycete infections. PLoS Pathog..

[57] Henshel D.S., DeWitt J., Troutman A. (2002). Using chicken embryos for teratology studies. Curr. Protoc. Toxicol..

[58] Burt D.W., Bruley C., Dunn I.C., Jones C.T., Ramage A., Law A.S., Morrice D.R., Paton I.R., Smith J., Windsor D. (1999). The dynamics of chromosome evolution in birds and mammals. Nature.

[59] Yaldiz C., Ceylan D., Sayin M., Kaçira T., Dilek F.H. (2015). The effects of levetiracetam on neural tube development of chick embryos. Neurosurg. Q..

[60] Ertekin T., Bilir A., Aslan E., Koca B., Turamanlar O., Ertekin A., Albay S. (2019). The effect of diclofenac sodium on neural tube development in the early stage of chick embryos. Folia Morphol..

[61] Chapman S.C., Collignon J., Schoenwolf G.C., Lumsden A. (2001). Improved method for chick whole-embryo culture using a filter paper carrier. Dev. Dyn..

[62] Henrique D., Adam J., Myat A., Chitnis A., Lewis J., Ish-Horowicz D. (1995). Expression of a Delta homologue in prospective neurons in the chick. Nature.

[63] Jiao Y.H., Dou M., Wang G., Li H.Y., Liu J.S., Yang X.X., Yang W.D. (2017). Exposure of okadaic acid alters the angiogenesis in developing chick embryos. Toxicon.

[64] Hellemans J., Mortier G., De Paepe A., Speleman F., Vandesompele J. (2007). qBase relative quantification framework and software for management and automated analysis of real-time quantitative PCR data. Genome Biol..

[65] Bustin S.A., Benes V., Garson J.A., Hellemans J., Huggett J., Kubista M., Mueller R., Nolan T., Pfaffl M.W., Shipley G.L. (2009). The MIQE guidelines: Minimum information for publication of quantitative real-time PCR experiments. Clin. Chem..

[66] Bolger A.M., Lohse M., Usadel B. (2014). Trimmomatic: A flexible trimmer for Illumina sequence data. Bioinformatics.

[67] Li B., Dewey C.N. (2011). RSEM: Accurate transcript quantification from RNA-Seq data with or without a reference genome. BMC Bioinform..

[68] Wang L., Feng Z., Wang X., Wang X., Zhang X. (2010). DEGseq: An R package for identifying differentially expressed genes from RNA-seq data. Bioinformatics.

[69] Conesa A., Götz S., García-Gómez J.M., Terol J., Talón M., Robles M. (2005). Blast2GO: A universal tool for annotation, visualization and analysis in functional genomics research. Bioinformatics.

[70] Abdi H. (2007). Bonferroni and Šidák corrections for multiple comparisons. Encyclopedia of Measurement and Statistics.

