# Supplementary material for: Okadaic Acid Exposure Induced Neural Tube Defects in Chicken (Gallus gallus) Embryos
Source: Mar Drugs. 2021 Jun 2;19(6):322. doi: 10.3390/md19060322 (PMC8227060; doi:10.3390/md19060322)
Supplement: Supplementary file 1 [file marinedrugs-19-00322-s001.zip › marinedrugs-1177225-supplementary.pdf]

Supplementary Table S1 Reads quality statistics

Sample	Total Raw Reads (M)	Total Clean Reads (M)	Total Clean Bases (Gb)	Clean Reads Q20 (%)	Clean Reads Q30 (%)	Clean Reads Ratio (%)
Ctr1	75.2	70.8	7.08	97.53	89.62	94.15
Ctr2	77.7	72.83	7.28	97.56	89.75	93.73
Ctr3	72.33	67.85	6.79	97.56	89.75	93.81
OA1	70.18	65.93	6.59	97.53	89.66	93.93
OA2	75.2	70.66	7.07	97.54	89.6	93.97
OA3	72.69	68.39	6.84	97.58	89.75	94.09

Supplementary Table S2 Reference genome alignment statistics

Sample	Total Clean Reads (M)	Total Mapping (%)	Uniquely Mapping (%)
Ctr1	70.8	89.96	67.82
Ctr2	72.83	89.84	67.97
Ctr3	67.85	89.6	67.81
OA1	65.93	89.27	67.17
OA2	70.66	90.16	68.03
OA3	68.39	89.5	67.5

Figure Legends

Supplementary Figure 1 Classification map of GO annotation after OA treatment.

Supplementary Figure 2 Differential gene KEGG Pathway classification after exposed to OA.

Supplementary Figure 3 Correlation between different biological replicates.

Figure S1

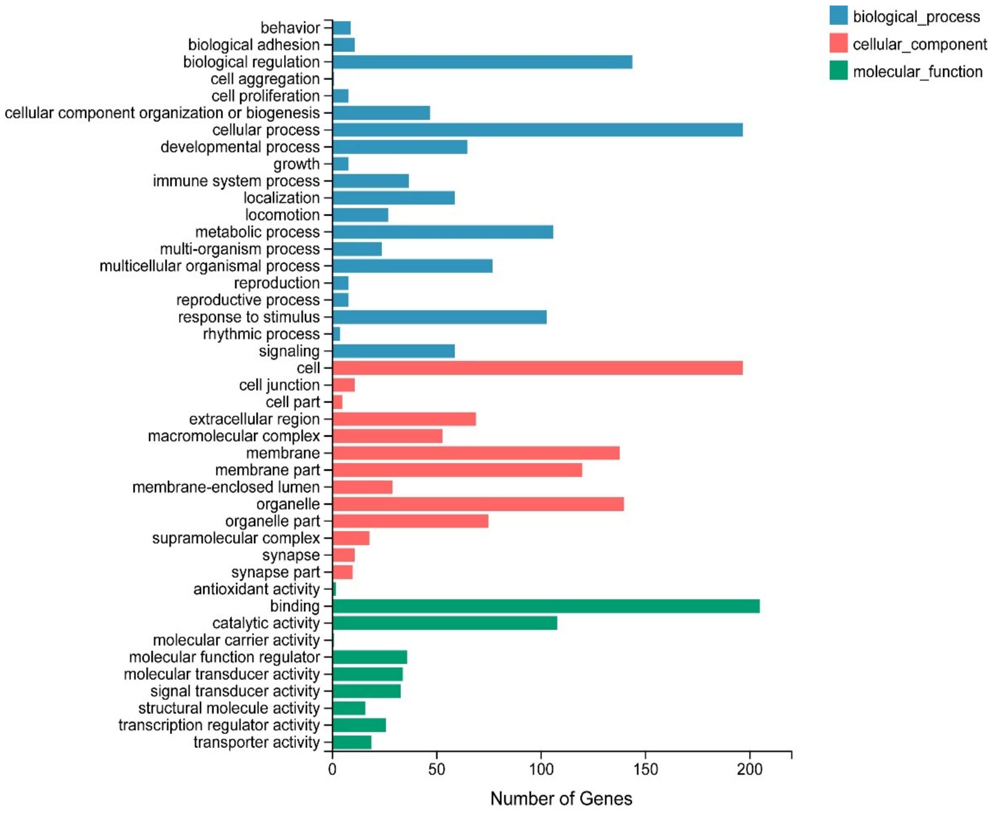
biological_process
cellular_component
molecular_function
behavior
biological adhesion
biological regulation
cell aggregation
cell proliferation
cellular component organization or biogenesis
cellular process
developmental process
growth
immune system process
localization
locomotion
metabolic process
multi-organism process
multicellular organismal process
reproduction
reproductive process
response to stimulus
rhythmic process
signaling
cell
cell junction
cell part
extracellular region
macromolecular complex
membrane
membrane part
membrane-enclosed lumen
organelle
organelle part
supramolecular complex
synapse
synapse part
antioxidant activity
binding
catalytic activity
molecular carrier activity
molecular function regulator
molecular transducer activity
signal transducer activity
structural molecule activity
transcription regulator activity
transporter activity
0
50
100
150
200
Number of Genes

Figure S2

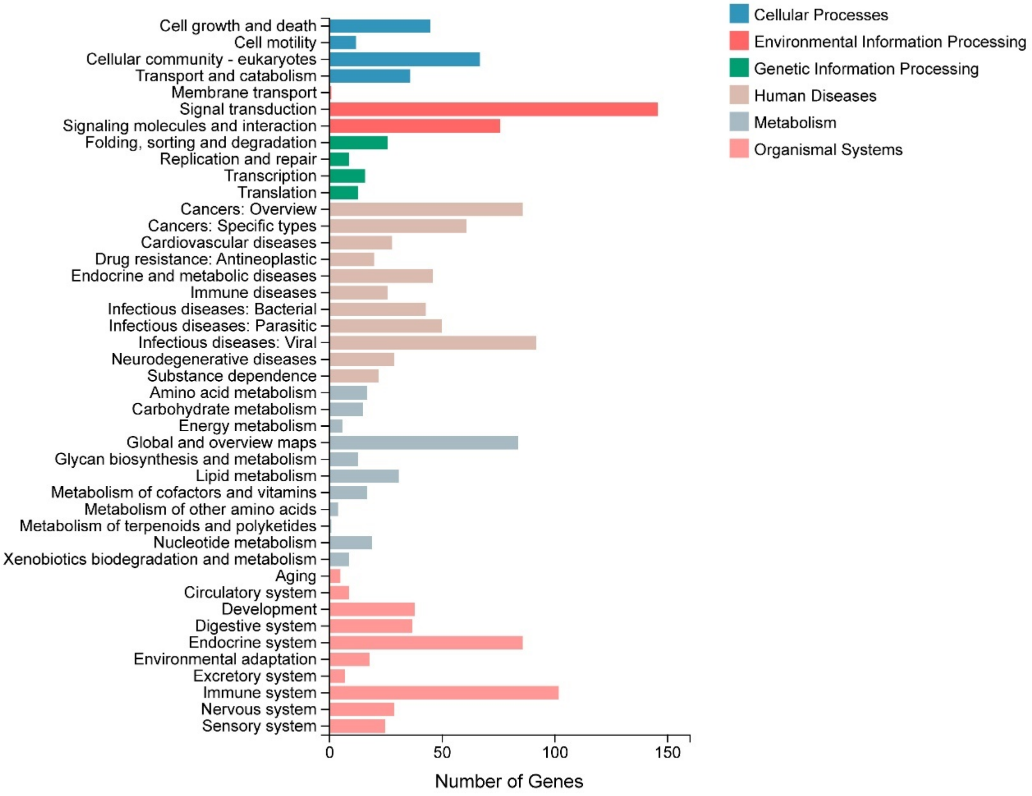

Cell growth and death
Cell motility
Cellular community - eukaryotes
Transport and catabolism
Membrane transport
Signal transduction
Signaling molecules and interaction
Folding, sorting and degradation
Replication and repair
Transcription
Translation
Cancers: Overview
Cancers: Specific types
Cardiovascular diseases
Drug resistance: Antineoplastic
Endocrine and metabolic diseases
Immune diseases
Infectious diseases: Bacterial
Infectious diseases: Parasitic
Infectious diseases: Viral
Neurodegenerative diseases
Substance dependence
Amino acid metabolism
Carbohydrate metabolism
Energy metabolism
Global and overview maps
Glycan biosynthesis and metabolism
Lipid metabolism
Metabolism of cofactors and vitamins
Metabolism of other amino acids
Metabolism of terpenoids and polyketides
Nucleotide metabolism
Xenobiotics biodegradation and metabolism
Aging
Circulatory system
Development
Digestive system
Endocrine system
Environmental adaptation
Excretory system
Immune system
Nervous system
Sensory system
0
50
100
150
Number of Genes
Cellular Processes
Environmental Information Processing
Genetic Information Processing
Human Diseases
Metabolism
Organismal Systems

Figure S3

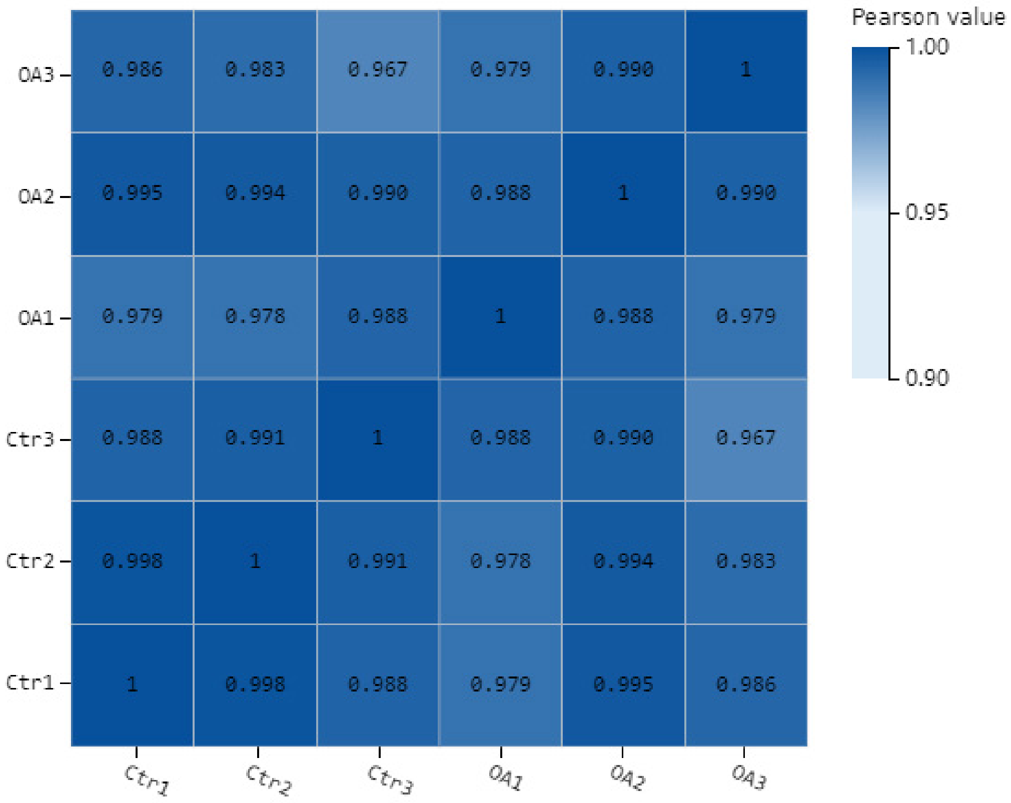
Pearson value
1.00
0.95
0.90
OA3
OA2
OA1
Ctr3
Ctr2
Ctr1
Ctr1
Ctr2
Ctr3
OA1
OA2
OA3
0.986 0.983 0.967 0.979 0.990 1
0.995 0.994 0.990 0.988 1 0.990
0.979 0.978 0.988 1 0.988 0.979
0.988 0.991 1 0.988 0.990 0.967
0.998 1 0.991 0.978 0.994 0.983
1 0.998 0.988 0.979 0.995 0.986
